# Gα_12/13_ signaling in metabolic diseases

**DOI:** 10.1038/s12276-020-0454-5

**Published:** 2020-06-23

**Authors:** Yoon Mee Yang, Da-Sol Kuen, Yeonseok Chung, Hitoshi Kurose, Sang Geon Kim

**Affiliations:** 1 College of Pharmacy, Kangwon National University, Chuncheon, 24341 South Korea; 2 College of Pharmacy, Seoul National University, Seoul, 08826 South Korea; 3 Department of Pharmacology and Toxicology, Graduate School of Pharmaceutical Sciences, Kyushu University, Fukuoka, 812-8582 Japan

**Keywords:** Mechanisms of disease, Metabolic disorders

## Abstract

As the key governors of diverse physiological processes, G protein-coupled receptors (GPCRs) have drawn attention as primary targets for several diseases, including diabetes and cardiovascular disease. Heterotrimeric G proteins converge signals from ~800 members of the GPCR family. Among the members of the G protein α family, the Gα_12_ family members comprising Gα_12_ and Gα_13_ have been referred to as *gep* oncogenes. Gα_12/13_ levels are altered in metabolic organs, including the liver and muscles, in metabolic diseases. The roles of Gα_12/13_ in metabolic diseases have been investigated. In this review, we highlight findings demonstrating Gα_12/13_ amplifying or dampening regulators of phenotype changes. We discuss the molecular basis of G protein biology in the context of posttranslational modifications to heterotrimeric G proteins and the cell signaling axis. We also highlight findings providing insights into the organ-specific, metabolic and pathological roles of G proteins in changes associated with specific cells, energy homeostasis, glucose metabolism, liver fibrosis and the immune and cardiovascular systems. This review summarizes the currently available knowledge on the importance of Gα_12/13_ in the physiology and pathogenesis of metabolic diseases, which is presented according to the basic understanding of their metabolic actions and underlying cellular and molecular bases.

## Metabolic diseases: Going after G proteins

Understanding the activities of two members of a vital category of proteins called G proteins, which initiate metabolic changes when signaling molecules bind to cells, could lead to new therapies for many diseases. Researchers in South Korea and Japan, led by Sang Geon Kim at Seoul National University, review the significance of the Gα12 and Gα13 proteins in diseases characterised by significant changes in metabolism, including liver conditions and disorders of the cardiovascular and immune systems. Specific roles for the proteins have been identified by a variety of methods, including studying the effect of disabling the genes that code for them in mice. Recent insights suggest that drugs interfering with the activity of these Gα proteins might help treat many conditions in which the molecular signalling networks involving the proteins are disrupted.

## GPCR–G protein pathways in metabolic diseases

Metabolic syndrome has been a serious health issue in the 21st century. It is a cluster of risk factors that can lead to cardiovascular diseases, diabetes, and stroke. Insulin resistance is the major factor that induces metabolic syndrome. It has been estimated that 642 million people will have type 2 diabetes by 2040^1^. The G protein-coupled receptor (GPCR) family is the largest membrane receptor family and serves as an attractive drug target. Currently, FDA-approved drugs, which account for approximately one-third of all drugs, target more than 100 GPCRs^2^. The glucagon-like peptide-1 (GLP-1) receptor, a member of the glucagon receptor family of GPCRs, is a metabolic syndrome-associated drug target. GLP-1 receptor agonists are used for glycemic control in patients with type 2 diabetes mellitus^3^. Because of its weight-loss effects, liraglutide (Saxenda^TM^) has become the first GLP-1 receptor agonist approved for the treatment of obesity^4^. Many GPCRs are pivotal sensors of energy metabolism. Some GPCRs are activated by energy metabolites or substrates, such as fatty acids, nucleotides, saccharides, hydroxycarboxylic acids, and citric acid cycle intermediates^5^. GPCRs activated by fatty acid-derived lipids have been proposed as antidiabetic drugs^6^. For example, the beneficial effect of omega-3 fatty acids is mediated by GPR120, also known as free fatty acid receptor 4. GPR120 is an attractive therapeutic target for the treatment of type 2 diabetes. GPR120-selective agonists improve insulin resistance and chronic inflammation and are currently under investigation for possible drug development^7^. Consequently, GPCR modulators are being actively investigated for their clinical applications in metabolic syndrome.

G proteins are recognized and activated by agonist-bound GPCRs. Heterotrimeric G proteins comprise the Gα, Gβ, and Gγ subunits. Gα mainly determines the functions of a G protein via the exchange of guanosine diphosphate (GDP) with guanosine triphosphate (GTP). The Gβ and Gγ subunits are synthesized separately and form a complex to function as a single functional unit. Both Gα and Gβγ subunits activate signaling molecules, thus eliciting cellular responses. Based on the characteristics of the Gα subunit, heterotrimeric G proteins are classified into four families: Gα_s_ (s represents stimulation), Gα_i_ (i represents inhibition), Gα_q_ and Gα_12_ families. The Gα_s_ family comprises Gα_s_ and Gα_olf_. The Gα_i_ family includes Gα_i1_, Gα_i2_, Gα_i3_, transducin-rod, transducin-cone, Gα_o-A_, Gα_o-B_, and Gα_z_. The Gα_q_ family includes Gα_q_, Gα_11_, Gα_14_, Gα_15_ (Gα_15_ is the mouse ortholog of Gα_16_) and Gα_16_. The Gα_12_ family comprises only two members: Gα_12_ and Gα_13_. Gβ comprises five members, and Gγ comprises 12 subtypes^8^. Thus, many combinations of Gα, Gβ, and Gγ subunits of heterotrimeric G proteins exist.

G protein signaling regulates the complexity of diverging and converging signal transduction systems. Thus, G protein levels may have a significant effect on the suppression or amplification of physiological and biochemical activities. The roles of Gα_i_ and Gα_q_ in metabolite-sensing signaling pathways have been extensively demonstrated. For example, Gα_i_ is coupled with the sweet taste receptor TAS1R2/TAS1R3 or with hydroxycarboxylic acid receptors^9^. Glucose, lactose, fructose, maltose, and sucrose are the ligands of TAS1R2/TAS1R3^10^. Hydroxycarboxylic acid receptors are activated by lactate, 3-hydroxybutyrate, and 3-hydroxyoctanoate^9^. Gα_q_ is the primary transducer of fatty acid receptors and citric acid cycle intermediate receptors. Some receptors, such as GPR43 and GPR91, utilize both Gα_i_ and Gα_q_^9^. Gα_s_-coupled receptors play roles in glucose and lipid metabolism. For instance, Gα_s_-coupled GPR119 regulates GLP-1 secretion in enteroendocrine L cells and insulin secretion in pancreatic β cells^11^. Gα_s_-coupled β-adrenergic receptors stimulate lipolysis in adipocytes.

Gα_12/13_, also known as *gep* proto-oncogenes, are often overexpressed in cancers, including breast^12^, prostate^13^, and liver^14^ cancers. They serve as prognostic factors, and high expression of Gα_12/13_ is associated with poor prognosis for patients^15,16^. The members of the Gα_12/13_ family have been recently identified as direct or indirect regulators of systemic energy metabolism. In this review, we summarize the posttranslational modifications of Gα_12/13_, the associated signaling pathways and the currently available knowledge on the pathophysiology of the G protein family with respect to energy metabolism and metabolic diseases.

## Posttranslational modifications of heterotrimeric G proteins

The Gα and Gγ subunits are posttranslationally modified by lipids. Lipid modification is essential for the functional maturation of G proteins. The functions of G proteins are also affected by phosphorylation at serine, threonine, and tyrosine residues. In addition to lipid modification and phosphorylation, G protein levels are affected by changes in stability (e.g., enhanced degradation) and increased transcription. Each topic has been described in detail in the following Section.

### Lipid modifications

The functional maturation of G proteins requires posttranslational modifications. The Gα subunit is modified by palmitoylation and myristoylation^17^. Myristoylation occurs at Gly in the amino-terminal domain, whereas palmitoylation occurs in the Gα subunit via thioesterification of the Cys residue. Gα_12/13_ undergo palmitoylation but not amidical myristoylation^18^. Palmitoylation of Gα_13_ is necessary for plasma membrane localization, Rho-dependent signaling, and the redistribution of a direct effector, p115-RhoGEF, from the cytoplasm to the plasma membrane^19^. Gα_12_ resides in lipid rafts, but Gα_13_ has not been found in lipid rafts. Prevention of Gα_12_ palmitoylation by mutating Cys-11 in Gα_12_ partially relocalized Gα_12_ away from lipid rafts^20^. Hsp90 specifically interacts with Gα_12_ but not with Gα_13_. Hsp90 interactions and the acylation of Gα_12_ facilitate Gα_12_ movement to lipid rafts^20^. Receptor stimulation induces the translocation of Gα_s_ from the plasma membrane to the cytoplasm. This translocation involves Gα_s_ depalmitoylation, which renders Gα_s_ less hydrophobic. However, opposite results have been reported by other research groups^21^. Thus, the role of depalmitoylation in membrane anchoring remains to be elucidated.

Palmitoylation is a reversible reaction; thus, receptor stimulation increases the turnover of palmitate. For instance, the turnover of palmitate attached to Gα_s_ is faster than the turnover of Gα_s_ itself. Isoproterenol stimulation of the β-adrenergic receptor and cholera toxin treatment increase the turnover rate without changing the total amount of palmitoylated Gα_s_^22^. Although palmitoylation is not the primary factor for the translocation of Gα_s_ to the plasma membrane, the palmitoylation-defective mutant of Gα_s_ is not associated with the membrane and is poorly coupled with adenylyl cyclase^23^. In contrast, Gα_q_ palmitoylation is essential for the stimulation of phospholipase C^23^. Thus, there is no consensus showing that the palmitoyl group is directly involved in the interaction of Gα with its effector molecules. Palmitoylation and depalmitoylation enzymes have been identified; however, their regulatory mechanisms remain to be elucidated.

### Phosphorylation

Gα subunits are phosphorylated by not only serine and threonine kinases but also tyrosine kinases. The Gβ subunit is phosphorylated by a kinase; however, this phosphorylation occurs on a His residue, and Gβ is an intermediate for the transfer of the phosphate group to GDP in the guanine nucleotide-binding pocket of the Gα subunit.

#### Phosphorylation of Gα subunits by PKA and PKC

Gα subunits are phosphorylated by kinases such as PKA and PKC, which are activated downstream of Gα_s_ and Gα_q_. PKA, stimulated by a membrane-permeable cAMP analog, phosphorylates Gα_13_. The PKA phosphorylation site of Gα_13_ is tentatively assigned to Thr203. Replacement of Thr203 with Ala results in a mutant with reduced affinity for Gβγ and decreased activation of RhoA^24^. This phosphorylation site is conserved in Gα_12_, another Gα_12/13_ family member, but not in other G protein families such as Gα_s_, Gα_i_, and Gα_q_. However, most Gα subunits contain PKA phosphorylation sites other than Thr203. Whether PKA phosphorylates Gα_s_, Gα_i_ and/or Gα_q_ to modulate their functions remains to be elucidated.

During platelet activation, Gα_12_ and Gα_13_ are phosphorylated^25^. Treatment with thrombin and phorbol 12-myristate 13-acetate phosphorylates Gα_12_ and Gα_13_ in a PKC-dependent manner. Among PKC subtypes, PKCβ, PKCδ, and PKCε efficiently phosphorylate Gα_12_ and Gα_13_^25^. Gα_12_ is also phosphorylated by other isoforms of PKC, including PKCα and PKCζ^26–28^. Phosphorylation enhances Gα_12_ activation, as in the case of Gα_z_.

PKC also phosphorylates other G proteins (e.g., the Gα_i_ and Gα_q_ family). In hepatocytes, the phosphorylation of Gα_i2_ is enhanced by phorbol esters that activate PKC^29^. Insulin treatment inhibits the basal and phorbol ester-stimulated phosphorylation of Gα_i2_, thereby increasing the nonphosphorylated active form of Gα_i2_. The increased phosphorylation of Gα_i2_ by PKC explains adenylyl cyclase inhibition upon insulin treatment. The phosphorylation of Gα_i2_ at Ser44, Ser144, and Ser302 by PKC promotes morphine-induced desensitization of the mu-opioid receptor^30^. PKC may be critical for the phosphorylation of Gα_11_ at Ser154, thereby inhibiting agonist-stimulated phospholipase C activity^31^.

5-HT_2A_ receptor stimulation induces the phosphorylation of Gα_q/11_ at Ser154. The phosphorylation of the 5-HT_2A_ receptor is mediated by PKC, decreasing this receptor coupling with Gα_q/11_^31^. 5-HT_2A_ receptors couple with Gα_q/11_, which implies a possible desensitization mechanism for the 5-HT_2A_ receptor.

PKC phosphorylates the Gα_z_ and Gα_q_ families. Phosphorylation of Gα_z_ has been demonstrated in vitro and in permeabilized platelets^32^. Phosphorylation at Ser16 inhibits the binding of Gβγ. As Gβγ binding is inhibited, Gα_z_ function is prolonged compared to that of the nonphosphorylated form.

PKC also phosphorylates Gα_15_, an ortholog of human Gα_16_, at Ser336. This phosphorylation site is located where the receptor and G protein interact. Thus, the phosphorylation-defective mutant of Gα_15_ is not activated by the receptor.

PAK, a p21-activated protein kinase, phosphorylates Gα_z_ at Ser16. PAK-promoted phosphorylation of Gα_z_ inhibits Gβγ binding, indicating that phosphorylation at the amino-terminal region regulates Gβγ binding^33^. This failure to bind leads to prolonged activation of Gα functions. The pathogenic bacterium *Yersinia* secretes Yersinia protein kinase A (YpkA), a Ser/Thr kinase, which phosphorylates Gα_q_ and inhibits GTP binding and Gαq-mediated signaling, implicating Gα_q_ phosphorylation by *Yersinia* as the cause of disease^34^.

#### Phosphorylation of Gα subunits by tyrosine kinases

The Gα subunit is phosphorylated by tyrosine kinases. Tyrosine phosphorylation of the Gα_q_ family occurs via receptor stimulation. M1 muscarinic acetylcholine receptor stimulation increases the tyrosine phosphorylation of Gα_11_ at Tyr356^35^; this phosphorylation site is located in the receptor recognition region, and its phosphorylation increases basal phospholipase C activity in vitro. This outcome suggests that Gα_11_ phosphorylation plays a role not only in receptor coupling but also in effector activation. Another study showed that coexpression of the M1 muscarinic acetylcholine receptor with the tyrosine kinase Fyn enhances Gα_q/11_ signaling.

Stimulation of metabotropic glutamate receptor 1α (mGluR1α) by glutamine increases the Tyr phosphorylation of cellular proteins, which is inhibited by the protein tyrosine kinase inhibitors genistein and tyrphostin AG213^35^. Glutamate stimulation-induced inositol-triphosphate production is inhibited by genistein and AG213, supporting the idea that tyrosine phosphorylation occurs prior to the action of phospholipase C.

Gα_s_ is phosphorylated by the proto-oncogene pp60^c-src^ at Tyr37 and Tyr377^36^. The phosphorylation of Gα_s_ stimulates GTPγS binding and receptor-stimulated GTPase activity. Because Y377 is located in receptor-coupling regions, such as Tyr356 in Gα_11_, pp60^c-src^-stimulated phosphorylation may affect receptor coupling. Hence, Src-mediated Gα_s_ phosphorylation may be critical for cancer-associated cell growth.

The EGF receptor stimulates tyrosine phosphorylation of Gα_s_, increasing Gα_s_-mediated adenylyl cyclase activity^37^. The insulin receptor directly or indirectly phosphorylates Gα_o_ and Gα_i_, although the functional consequences have not yet been demonstrated. It remains unknown whether Gα_i_, Gα_q_ or Gα_12_ family G proteins are phosphorylated by tyrosine kinases.

### Degradation

The expression of Gα_12/13_ is often deregulated in metabolic diseases. For example, hepatic Gα_12_ expression is decreased in humans with nonalcoholic fatty liver disease^38^. Hepatic Gα_13_ is downregulated^39^, whereas Gα_13_ is overexpressed in the skeletal muscle of patients with diabetes^40^. These findings suggest that the balance between Gα_12/13_ synthesis and degradation is disrupted under pathological conditions. However, the molecular mechanism underlying Gα_12/13_ degradation has not yet been fully elucidated. Here, we review the degradation process of other Gα proteins.

#### Ubiquitination of Gα subunits

Cholera toxin catalyzes the ADP-ribosylation of Arg201, which is critical for the GTP-hydrolyzing activity of Gα_s_. Because ADP-ribosylation inhibits GTPase activity, Gα_s_ is activated even without receptor stimulation. Treatment with the cholera toxin increases the Gα_s_ level in the cytoplasm and enhances its degradation rate. This finding suggests that Gα_s_ is degraded after being dissociated into Gα and Gβγ. Compared to GDP-bound inactive Gα_s_, in the cytoplasm, GTP-bound active Gα_s_ is subjected to an efficient degradation system^41^. However, it remains unknown whether Gα_s_ degradation is mediated completely by the ubiquitin-proteasome system. The precise degradation mechanism remains to be determined.

#### Regulation by Ric-8

There is no definitive evidence supporting the idea that the G protein undergoes ubiquitin-mediated regulation. However, accessary G proteins were found to be controlled in a ubiquitin-dependent manner. Ric-8 was originally found to positively regulate neurotransmitter release through Gα_q_ in *Caenorhabditis elegans*. Contrary to the *C. elegans* and *Drosophila* genomes, the mammalian genome contains two members of Ric-8 (Ric-8A and Ric-8B)^42^.

Ric-8A functions as the guanine nucleotide exchange factor (GEF) associated with Gα_q_, and knocking down Ric-8A by siRNA inhibits ERK activation and intracellular Ca^2+^ increase^42^. In Ric-8A-knockout fibroblasts, the protein levels of Gα_13_ and other G proteins such as Gα_i1-2_, Gα_o_, and Gα_q_ were decreased to 10% those in wild-type cells. In contrast to the Ric-8A-knockout cells, Ric-8B knockout cells exhibited only reduced levels of Gα_s_^42^. Ric-8B interacts with Gα_olf_, Gα_s_, and Gα_q_. The coexpression of Ric-8B and Gα_olf_, a Gα_s_ family member, with dopamine D1 and β2-adrenergic receptors enhanced receptor-stimulated cAMP production in HEK293 cells. In NIH-3T3 cells, knocking down Ric-8B inhibited isoproterenol-stimulated cAMP production and decreased the expression level of Gα_s_^42^. However, it did not affect the expression levels of other Gα proteins, such as Gα_i_ and Gα_q_. The suppression of Ric-8B expression did not affect Gα_s_ mRNA expression. Thus, the function of Ric-8B is to stabilize the Gα_s_ protein.

The Ric-8 protein interacts with Gα, thus stabilizing the Gα subunit. Gα is stabilized via the inhibition of ubiquitination^43^. Although the E3 ubiquitin ligase for Gα proteins has not yet been identified, Ric-8 has been reported to inhibit ubiquitination and stabilize Gα proteins. Because Ric-8 controls the level of the Gα subunit, ubiquitin-mediated Ric-8 regulation is critical to G protein levels and G protein-mediated responses. Although the mechanism by which Ric-8 expression is regulated has not been reported in detail, receptor stimulation both increases and decreases the expression level of Ric-8^42^. Thus, Ric-8^4^ may be part of a newly discovered type of regulatory mechanism that modulates receptor-mediated signaling through G proteins. Ubiquitination regulates G protein levels in rod photoreceptors, with light-dependent translocation of transducin between the outer and inner photoreceptor segments important for light/dark adaptation and the prevention of cell damage by light. Inhibition of transducin α subunit (Tα) translocation from the inner part to the outer segment reduces light responses. In contrast, Tα is translocated to the outer segment to enhance light reception sensitivity. Cullin 3 (Cul3)-Kelch-like 18 (Klhl18) ubiquitin E3 ligase modulates the translocation of Tα^44^. Cul3-Klhl18 ubiquitinates UNC119, a Tα-interacting protein, and promotes the degradation of UNC119. UNC119 is a lipid-binding protein that interacts with the acylated amino-terminus of Tα. Casein kinase 2 phosphorylates UNC119, and this phosphorylation inhibits its degradation by Cul3-Klhl18. Thus, ubiquitination indirectly modulates the location and expression of Tα in rod photoreceptors.

### Other regulatory mechanisms mediated by Gα subunits

Promoter analysis and the transcriptional regulation of G proteins via transcription factors have not been completely characterized to date. However, the posttranscriptional regulation of Gα_12/13_ by microRNA has been reported. Kim et al.^45^ found the reciprocal expression of miR-16 and Gα_12_ in liver fibrosis. miR-16 is abundantly expressed in quiescent hepatic stellate cells (HSCs), and its expression is diminished in activated HSCs. miR-16 targets Gα_12._ Dysregulation of miR-16 contributes to liver fibrosis by facilitating Gα_12_-mediated autophagy in HSCs. miR-31 and miR-30d directly target Gα_13_ and inhibit the invasion of breast cancer cells and colorectal cancer cells, respectively^46,47^. miR-182 and miR-141/200a regulate Gα_13_ posttranscriptionally^48^.

## The biology of GPCRs coupled to Gα_12/13_

Gα_12/13_ transduce signals from more than 30 GPCRs. Lysophosphatidic acid receptors (LPA), sphingosine-1-phosphate receptors (S1P1–S1P5), angiotensin II type 1 receptors (AT1) and thrombin receptors (PAR1) couple with Gα_12/13_^49,50^ (Table 1). In response to ligands, the conformation of GDP-bound inactive Gα_12/13_ is transformed to the GTP-bound active form. The guanine nucleotide-dependent conformational change of G proteins results in the release of GTP-bound Gα_12/13_ from the Gβγ subunits. The Gα_12_ family members regulate RH-RhoGEF, which contains an RGS homology domain and activates GTPase Rho^51,52^. The RH-RhoGEF family contains p115RhoGEF, leukemia-associated RhoGEF (LARG) and PDZ-RhoGEF. P115RhoGEF and LARG exhibit GAP activity specifically toward Gα_12/13_^53^, whereas PDZ-RhoGEF does not affect Gα_12/13_. Gα_13_ directly stimulates p115RhosGEF and LARG^54,55^, and Gα_12/13_ are deactivated by the hydrolysis of GTP to GDP by intrinsic GTPase activity and GTPase-activating proteins (GAPs)^54–56^. Thus, Gα_12/13_ signaling is fine-tuned by RH-RhoGEF family.

**Table 1:** Gα_12/13_-associated GPCRs and physiological functions.

Receptors	G proteins	Functions	References
Sphingosine 1-phosphate			
S1P2/S1P3	Gα_12/13_	Stress fiber formation	^133^
S1P1/S1P3/S1P5	Gα_12_	Inflammation	^62^
S1P3	Gα_12/13_	Inflammation	^134^
S1P2	Gα_12/13_	Myofibroblast contraction	^135^
S1P3	Gα_13_	Cardioprotection	^136^
S1P receptor	Gα_12_	Hepatic stellate cell activation	^45^
Thrombin			
PAR1	Gα_12_	Monocyte migration	^63^
PAR1	Gα_13_	Cell transformation	^137^
PAR1	Gα_12/13_	Endothelial cell permeability	^138^
Thrombin receptor	Gα_12/13_	NO production in macrophage	^139^
Thrombin receptor	Gα_12_	Stress fiber accumulation	^140^
Thrombin and thromboxane A2	Gα_12/13_	Platelet activation	^25^
Lysophosphatidic acid			
LPA4	Gα_12/13_	Limits proper adipose tissue expansion and remodeling in diet-induced obesity	^141^
LPA4	Gα_12/13_	Hypertensive response	^122^
LPA4/LPA6	Gα_12/13_	Angiogenesis	^142^
LPA receptor	Gα_13_	Stress fiber formation	^140^
Angiotensin			
AT1R	Gα_12/13_	Hyperplasia of cardiac fibroblasts	^143^
AT1R	Gα_12/13_	Vascular endothelial dysfunction	^144^
Endothelin			
ET_A_	Gα_12_	Stress fiber accumulation	^140^
ATP			
P2Y6	Gα_12/13_	Cardiac fibrosis	^131^
Adenosine			
A1/A2a/A2b/A3	Gα_12_	Fatty acid oxidation	^38^
Bradykinin			
B2	Gα_13_	Stress fiber formation	^140^
Serotonin			
5-HT2C	Gα_13_	Stress fiber formation	^140^
Vasopressin			
V1A	Gα_12_	Stress fiber accumulation	^140^

Rho kinase (ROCK) is a downstream effector of the Gα_12/13_-RhoA signaling pathway, which in turn phosphorylates various substrates, such as MLC phosphatase, ERM, LIMK, diaphanous, rhotekin, rhophilin, citron kinase, and CRPM-2^57^. Both the RhoA inhibitor C3 toxin and ROCK inhibitor Y-27632 prevent Gα_12/13_-mediated RhoA- and ROCK-dependent MLC_20_ phosphorylation^58^. Gα_12/13_ also activate Jun kinase (JNK), which in turn phosphorylates Jun and ATF2^59–61^. Gα_12/13_ play roles in inflammatory responses. Sphingosine-1-phosphate, a sphingolipid produced by platelets, stimulates cyclooxygenase-2 (COX-2), which is mediated by the S1P1/S1P3/S1P5-Gα_12_ pathway^62^. Gα_12_ regulates sphingosine-1-phosphate-mediated NF-κB-mediated COX-2 induction, which depends on JNK-dependent ubiquitination and the degradation of IκBα^62^. Thrombin induces monocyte migration via PAR1-Gα_12_-p115RhoGEF activation through a Gα_12_-mediated pathway^63^. Gab1 interacts with p115RhoGEF and induces monocyte F-actin cytoskeletal remodeling and migration via Rac1- and RhoA-dependent Pak2 activation^63^. Therefore, Gα_12/13_-coupled receptor signaling plays a role in cell mobility, growth, differentiation, inflammation, and transcription^64^.

## Gα_12/13_ in the immune response

Inflammation is an extremely dynamic response mediated by interactions among various immune cells. Immune cell activation, migration to the site of inflammation and inflammatory effector function are largely facilitated by the coordination of various chemokine receptors that are structurally similar to GPCRs^65^. Examples of receptors known to interact with Gα_12/13_ proteins include chemokines, S1P, LPA, and thrombin receptors, which are important for recruiting immune cells to the site of inflammation and promoting entry into lymphoid organs, thereby amplifying the inflammatory response^66^. In addition to the abovementioned lipids and chemokines, protons can also act as Gα_12/13_ activators, and are highly abundant in lymphocytes. Dysregulation of Gα_12/13_ leads to lymphoid hyperactivation and/or malfunction. Mouse studies have shown that Gα_12/13_ disruption leads to lymphoid hypertrophy and adenopathy, demonstrating the importance of Gα proteins in fine-tuning immune responses^67,68^. Furthermore, dysregulation of Gα_12/13_ in immune cells may lead to pathophysiological consequences, such as cancer and autoimmunity^68–71^.

### Gα_12/13_ regulation in B cells

Gα_12_ may be involved in B cell maturation based on its binding and activation of BTK, a kinase required for normal B cell development and activation. The reduction in MZB precursors in B cell-specific Gα_12/13_‐deficient mice also suggests a role for Gα_12/13_ in peripheral MZB maturation^72^. The migration of B cells in response to serum and S1P treatment is highly increased in mutant MZB cells but not in follicular B cells, indicating that Gα_12/13_ family members contribute to the formation of mature MZB cells by controlling precursor migration^72^. Gα_12/13_-coupled receptors, such as S1PR2 and P2RY8, have been shown to regulate B cell confinement in the germinal centers within SLOs^69,73^, and loss-of-function mutations have been directly associated with oncogenic proliferation and the impaired apoptosis of germinal center B cells^68,69^. Despite the high expression of Gα_12/13_-mediated receptors, precise chemokine gradients that enable accurate B cell positioning and movement throughout various developmental stages have only recently been elucidated^74^.

### Gα_12/13_ regulation in T cells

Gα_13_‐mediated signaling, but not Gα_12_-mediated signaling, has been shown to be necessary for early thymocyte proliferation and survival^75^. The expression of a mutant form of p115RhoGEF, a Gα_13_ effector in progenitor T cells, leads to reduced proliferation and increased apoptosis rates at the double‐negative stage of T cell development. Accurate Gα_12/13_-mediated signaling has also been shown to be important to CD4 T cells in various stages after complete thymocyte development. Gα_12/13_-coupled receptors negatively regulate cell polarization and adhesion, thereby controlling effector T cell entry in and exit from secondary lymphoid organs^67^. T cells are activated via TCR signaling, and downstream cascade molecules have been shown to interact with Gα_13_ to mediate serum response factor transcriptional activity^76^. Following naive T cell activation, CD4^+^ T cells differentiate into effector subsets: Th1, Th2, Th17 and Tfh cells, and each of these subsets has a specified helper function that facilitates protection of the host against various types of foreign pathogens^77^. In particular, Tfh cells act as critical helper T cells that facilitate B cell maturation and antibody response^78^. Gα_12/13_-mediated receptor signaling is important in Tfh differentiation and functions by coordinating cell localization cues^79^. STAT3 activation has been suggested to be the key target of regulation by which Gα_12/13_-mediated signaling modulates inflammatory T cells such as Th17 cells^80,81^. ROCK2 has been specifically shown to directly interact with phosphorylated STAT3 and co-occupy Th17/Tfh gene promoter regions of key transcription factors such as Irf4 and Bcl6 in human Th17 cells^81^. ROCK2-specific inhibitor treatment led to lower levels of STAT3-mediated IL-17 and IL-21 cytokine secretions in T cells from both healthy and rheumatoid arthritis patients. In addition, ROCK inhibitor treatments in a mouse model of autoimmunity promoted STAT5-mediated Treg activity, thereby restoring disrupted immune homeostasis^80^. These findings indicate that the regulation of GPCR-mediated signaling in immune cells has important consequences in the context of allergy and autoimmunity in addition to its effects on metabolic diseases^70,71^.

## Roles of Gα_12/13_ in physiology and pathology

### Role of Gα_12/13_ in energy homeostasis

The liver is the key metabolic organ maintaining whole-body energy balance. The liver regulates carbohydrate, fat and protein metabolism. When excess glucose enters the blood circulation after a meal, insulin stimulates the liver to synthesize glycogen for storage. Upon a decrease in blood glucose content, glycogen is broken down into glucose through glycogenolysis. Excess carbohydrates can be converted into free fatty acids and triglycerides via hepatic de novo lipogenesis. Insulin autonomously regulates lipid synthesis in the liver^82^. Under insulin-resistant conditions, insulin continues to promote lipogenesis but fails to suppress hepatic glucose production^82^. In the catabolic pathway, the liver produces ATP via fatty acid oxidation, which is the mitochondrial aerobic process of fatty acid breakdown that generates acetyl-CoA. The liver also synthesizes nonessential amino acids and plasma proteins. When the fine-tuned regulation of energy balance is disrupted in the liver, nonalcoholic fatty liver disease can develop. A high-fat diet supplemented with fructose impairs hepatic mitochondrial function and decreases fatty acid oxidation, thereby contributing to nonalcoholic fatty liver disease^83^.

Hepatic expression of Gα_12_ is upregulated in the fasted state^38^. *Gna12*-KO mice had increased lipid accumulation in the liver after fasting than did the corresponding wild-type mice. The role of Gα_12_ in the regulation of mitochondrial respiration, as mediated by the SIRT1/PPARα network, was identified via microarray analyses and in vivo experiments^38^. SIRT1, an NAD^+^-dependent protein deacetylase, is an important regulator of the PPARα-mediated transcriptional network involved in fatty acid oxidation^84^. The Gα_12_ pathway governs mitochondrial respiration, lipid catabolism, acyl-CoA metabolism, ketogenesis and peroxisomal oxidation through SIRT1 stabilization^38^.

**Fig. 1:**
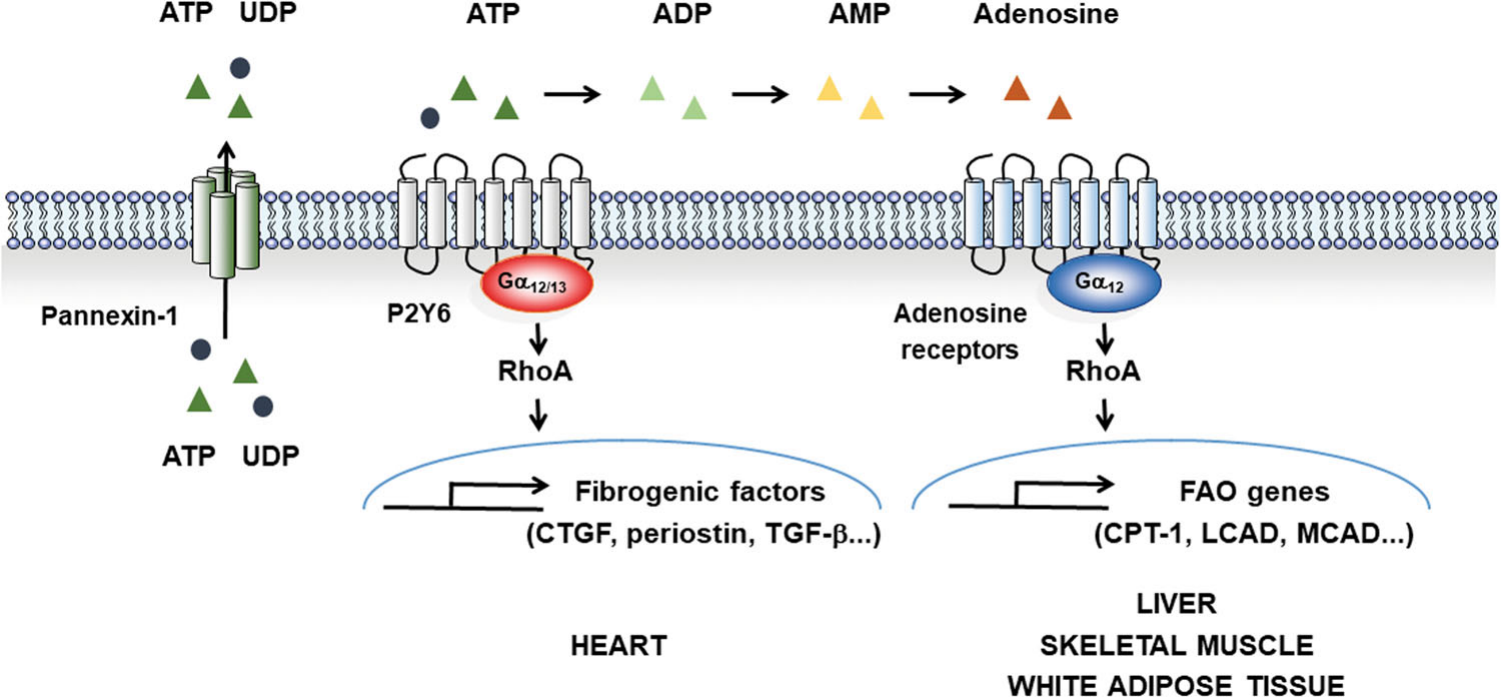
Schematic depiction of purinergic receptors coupled with Gα_12/13_ and physiological events. Intracellular ATP or UDP is released through Pannexin 1 channels. Extracellular ATP or UDP binding to the purinergic P2Y6 receptor in cardiomyocytes mediates mechanical stretch-induced Gα_12/13_ and Rho activation. P2Y6-Gα_12/13_ signaling is critical for mechanical stretch-induced fibrotic factors such as connective tissue growth factor (CTGF), periostin and transforming growth factor (TGF)-β. Extracellular ATP is actively broken down to ADP, AMP and, ultimately, adenosine by ectonucleotidases and may serve as GPCR ligands. Gα_12_ is involved in the fatty acid oxidation process downstream of adenosine receptors.

Fasted mice show increased serum adenosine concentrations. Adenosine is produced and released from most tissues. In addition, extracellular adenine nucleotides can be broken down into adenosine, which serves as a ligand for four distinct GPCRs (A_1_, A_2a_, A_2b_, and A_3_). Adenosine receptor agonists and antagonists have been tested for their effects against liver ischemia, liver cancer, cardiovascular disease, and Parkinson’s disease^85–87^. The adenosine receptor agonists stimulated SIRT1 expression through Gα_12_^38^ (Fig. 1). Gα_12_ stabilized SIRT1 protein through the HIF-1α-mediated transcriptional induction of ubiquitin-specific peptidase 22 (USP22)^38^. Therefore, high levels of Gα_12_ expression promote mitochondrial respiration. Compared with that in patients without steatosis, the Gα_12_ level in patients with NAFLD was diminished in the liver. Consequently, Gα_12_ deficiency results in high-fat diet-induced obesity and hepatic steatosis. Adenosine receptor-Gα_12_ coupling plays a role in lipid metabolism via the SIRT1/PPARα pathway. Gα_12_ is also found in mitochondria, and mitochondrial Gα_12_ is associated with decreased mitochondrial motility^88^. Gα_12_ binds to the inner surface of the cell membrane, and Gα_12_ specifically targeted to the mitochondria may control mitochondrial respiration, morphology, and dynamics in a distinct manner. During adipocyte hypertrophy and hyperplasia, white adipose tissue undergoes dynamic expansion and remodeling. The activated Gα_12_ mutant increased B-Raf and MEK1 expression and MAPK activity. Thus, the active form of Gα_12_ enhanced the proliferation of preadipocytes but prevented their differentiation^89^. Rho/ROCK, downstream from Gα_12/13_, inhibited 3T3-L1 adipogenesis in response to G-protein-deamidation of dermonecrotic toxins^90^. LPA4 receptors are exclusively expressed in epididymal white adipose tissue. Octadecenyl phosphate is an agonist of LPA4. The ODP–LPA4 axis activates Gα_12/13_ in adipocytes, and LPRA4 in adipocytes limits the continuous remodeling and healthy expansion of white adipose tissue via Gα_12/13_. LPA4 activation by octadecenyl phosphate treatment decreases PPARα-associated gene expression. Consistently, the loss of LPA4 promotes adipose tissue expansion and protects against high-fat diet-induced hepatic steatosis and insulin resistance^91^.

Skeletal muscle is one of the major organs in systemic energy homeostasis because it requires a high amount of nutrients. Mammalian skeletal muscles have two types of myofibers: oxidative and nonoxidative; these myofibers show distinct metabolic characteristics. Endurance exercise training reprograms myofiber types into oxidative fibers, which have a higher capacity for mitochondrial respiration and fatty acid oxidation^92^. Obese individuals or patients with type 2 diabetes possess fewer oxidative myofibers. GPR56 is an adhesion GPCR, and it transduces signals through activated Gα_12/13_-Rho. GPR56 is involved in mechanical overload-induced muscle hypertrophy^93^. PGC-1α4, an alternatively spliced form of PGC1α, transcriptionally regulates GPR56 induced by resistance exercise. Consistently, Gα_12_ and Gα_13_ mRNA levels increase in human subjects performing resistance training than in sedentary individuals.

**Fig. 2:**
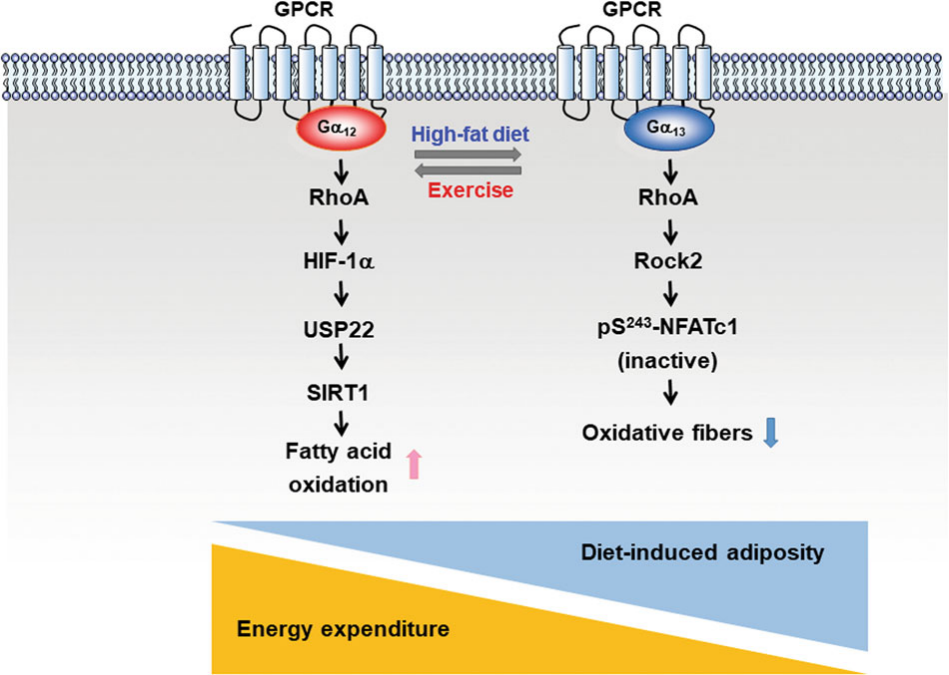
The roles of Gα_12_ and Gα_13_ are switched to regulate pathways for energy expenditure and high-fat diet-induced adiposity. Gα_12_ levels are lower in the liver of high-fat diet (HFD)-fed mice and in patients with steatosis and/or nonalcoholic steatohepatitis. Gα_12_ transduces signals of deubiquitination and stabilization of SIRT1 through HIF-1α-mediated transcriptional control of ubiquitin-specific peptidase 22 (USP22). SIRT1 governs the PPARα transcriptional network in metabolic processes, particularly fatty acid oxidation. The Gα_12_ pathway facilitates whole-body energy expenditure through USP22/SIRT1-regulated mitochondrial respiration. Gα_13_ levels in skeletal muscle are decreased in the exercise-induced state (a condition of energy deficiency) but are increased in mice fed an HFD or in patients with type 2 diabetes. Gα_13_-RhoA-ROCK2 phosphorylates nuclear factor of activated T cells 1 (NFATc1) at Ser243 to inhibit NFATc1 activation. NFATc1 contributes to the transformation of fibers into oxidative-type fibers. Deficiency of Gα_13_ in skeletal muscle promotes energy expenditure, thereby protecting mice from metabolic challenge induced by NFATc1-dependent myofiber-type reprogramming.

Studies investigating Gα_13_ have been limited because the gene-knockout animal model showed a significant defect in vasculogenesis during development, which led to embryo lethality^94^. Using the Cre–loxP system, muscle-specific Gα_13_-knockout mice were generated^40^. In this study, knocking out skeletal muscle-specific *Gna13* promoted the reprogramming of oxidative-type myofibers, with resultant increases in mitochondrial biogenesis. Gα_13_ and its effector RhoA suppressed NFATc1 by increasing Rock2 and was critical for the phosphorylation at Ser243 in NFATc1; this suppression and phosphorylation were reduced after exercise but were higher in HFD-fed obese animals. Consequently, the muscle-specific ablation of Gα_13_ increased whole-body energy metabolism, protecting animals from obesity and liver steatosis. In the absence of Gα_13_, Gα_12_ plays a role in mitochondrial regulation in skeletal muscle^38^. Thus, the Gα_12_ signaling pathway controls mitochondrial energy expenditure via SIRT1-mediated and HIF-1α-dependent USP22 induction^38^ (Fig. 2).

Even though both Gα_12_ and Gα_13_ commonly activate RhoA, the downstream effectors and functions are diverse among tissues. In the postprandial state, dietary triglycerides are transported to the liver from the intestines, and then, the liver utilizes fatty acids and glycerol to synthesize triglycerides. Excessive amounts of nutrients are mainly stored in fat and liver tissues. Obesity increases HIF-1α levels and decreases the sinusoidal blood flow rate and velocity in the liver^95^. Hypoxia enhances nonalcoholic fatty liver disease^96^. The inhibition of the RhoA/Rock pathway attenuated the effect of Gα_12_ overexpression on HIF-1α. Gα_13_ activates Rho/Rock2. Rock2 phosphorylates NFATc1 in skeletal muscle. NFATc1 is not expressed in the liver (https://www.proteinatlas.org/ENSG00000131196-NFATC1/tissue/liver). Therefore, we presume that Gα_12_ activates HIF-1α in the liver and that Gα_13_ inactivates NFAT1c in skeletal muscle.

### Role of Gα_12/13_ in glucose metabolism

Because insulin resistance causes diabetes and contributes to metabolic syndrome in multiple organs, approaches targeting single organs have limitations. The liver senses extracellular nutritional availability and regulates overall glucose metabolism. Sustained excessive intake of calories leads to fat accumulation and liver steatosis. Steatosis, frequently accompanied by hyperglycemia, usually leads to metabolic dysfunction in other organs^97–99^, suggesting a causal role of liver pathophysiology in the dysregulation of systemic energy homeostasis. The increased hepatocellular lipid content causes hepatic insulin resistance. Excessive free fatty acids and imbalanced adipocytokines cause not only insulin resistance but also promote the progression of hepatic steatosis to nonalcoholic steatohepatitis and cirrhosis. Hepatic fat content is a key determinant of metabolic flux in insulin resistance and type 2 diabetes mellitus^100^. Nevertheless, the evidence that the liver may be the origin and driver of the systemic disruption of energy metabolism primarily involving insulin resistance in the setting of metabolic disease progression has drawn little attention.

According to a phase III placebo-controlled study, fasiglifam (TAK-875), a partial GPR40 agonist, effectively lowers HbA1c in people with type 2 diabetes^101^. Due to off-target liver toxicity, the clinical development of fasiglifam was terminated^102^. Subsequently, GPR40 full agonists have been under development in preclinical settings. A GPR40 allosteric full agonist enhanced the glucose-stimulated insulin secretion in pancreatic β cells via the GPR40-mediated activation of Gα_12_^103^. However, the overexpression of Gα_12_ decreased insulin secretion through JNK^38^. *Gna12*-KO mice fed a HFD displayed lower fasting glucose levels with hyperinsulinemia. Nevertheless, whole-body glucose flux was not significantly altered by Gα_12_ deficiency.

**Fig. 3:**
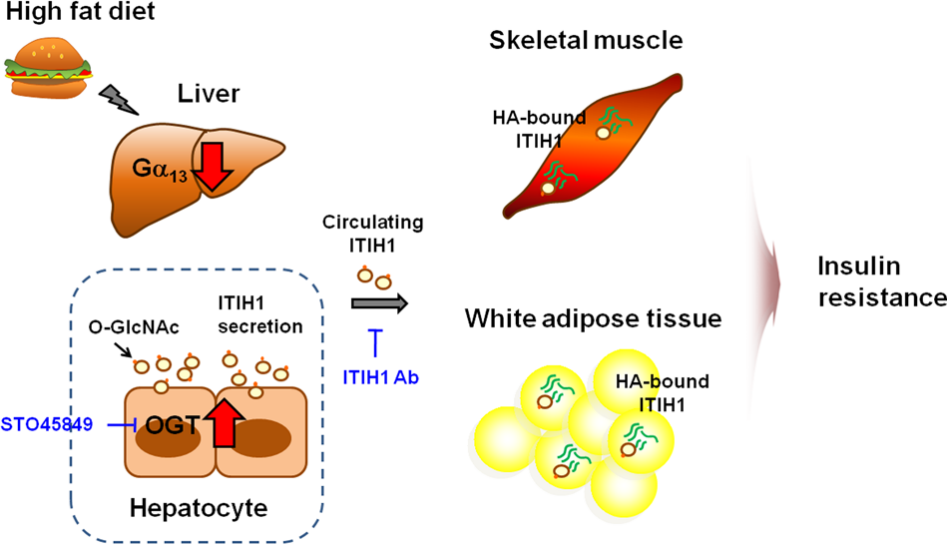
The role of Gα_13_ in interorgan biological processes in insulin resistance. Hepatic Gα_13_ levels are downregulated in high-fat diet (HFD)-fed or genetically obese mice and patients with diabetes. In response to a decrease in Gα_13_, the inter-α-trypsin inhibitor heavy chain 1 (ITIH1) is *O*-GlcNAcylated by *O*-GlucNAc transferase (OGT) induced in hepatocytes and then excessively secreted into the bloodstream. ITIH1 is a binding partner of HA, one of the major extracellular matrix components. In mice deficient in Gα_13_ in hepatocytes, increased ITIH1 is deposited onto the hyaluronan surrounding skeletal muscle and white adipose tissue. Overproduction of ITIH1 from the liver after the loss of Gα_13_ causes systemic insulin resistance. Treatment with ST045849 (an inhibitor of *O*-GlcNAC transferase) or antibody neutralization of ITIH1 ameliorates systemic insulin resistance.

Hyperglycemia decreases Gα_13_ in the liver, eventually contributing to glucose intolerance and insulin resistance in other metabolic organs by overproducing liver-secretory O-GlcNAc protein (Fig. 3)^39^. With HFD feeding, hepatocyte-specific Gα_13_-knockout mice exhibited exacerbated glucose tolerance and insulin resistance, although a normal diet had no effect on the metabolic phenotypes, such as body weight gain and fasting blood glucose content^39^. Therefore, the decrease in Gα_13_ in hepatocytes was clearly manifested by metabolic challenges and was distinctively associated with glucose utilization.

**Fig. 4:**
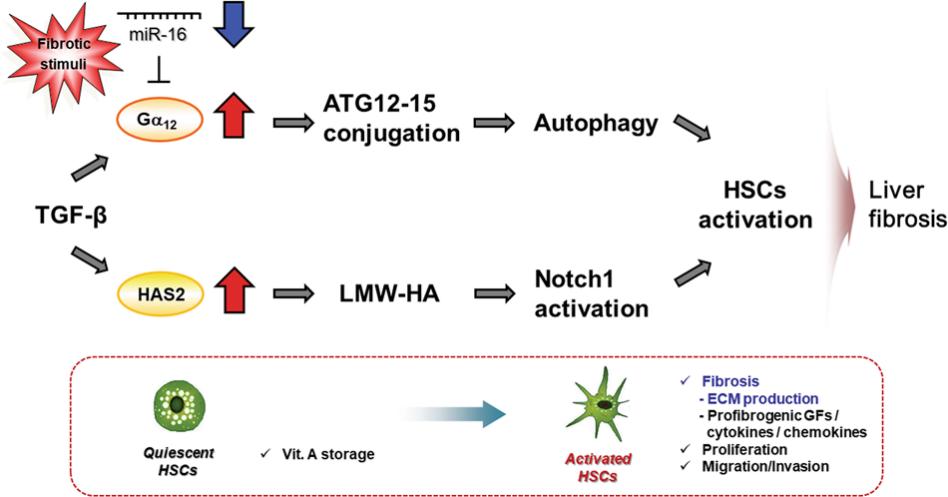
Gα_12_ overexpression in hepatic stellate cells during liver fibrosis. miR-16 directly targets Gα_12_. In activated hepatic stellate cells, miR-16 is dysregulated. Overexpression of Gα_12_ due to a decrease in miR-16 promotes autophagy through JNK-mediated ATG12-5 conjugation. The Gα_12_ signaling pathway contributes to hepatic stellate activation. In response to transforming growth factor-β (TGF-β), a central mediator of fibrogenesis, hyaluronan synthase 2 (HAS2) is transcriptionally upregulated. HAS2 synthesizes high-molecular-weight HA (HMW-HA). Reactive oxygen species or hyaluronan-degrading enzymes facilitate the conversion of HMW-HA into low molecular weight (LMW)-HA. LMW-HA treatment activates Notch1, which is critical for liver fibrosis.

The extracellular matrix (ECM) is a highly dynamic compartment consisting of different extracellular proteins. The ECM modulates not only biological processes, including cell growth and migration, but also physiological communication. Thus, ECM remodeling in peripheral tissues affects glucose metabolism and insulin signaling under diabetic conditions. A number of pathological conditions affect aberrant ECM remodeling and deposition. Notably, the stiffness or rigidity of the ECM also significantly affects cellular function and is highly dependent on various interacting proteins, stabilizing and potentiating their binding properties with other ECM proteins. Hyaluronan (HA) is one of the major components of the ECM, and its level is increased in insulin-resistant tissues. Excessive accumulation of HA in metabolic tissues has been observed in obese diabetic mice^104^. Moreover, serum HA levels are increased in patients with diabetes and liver fibrosis^105,106^. Depletion of HA via the intravenous administration of hyaluronidase in mice led to improvements in systemic glucose tolerance and insulin sensitivity, indicating the crucial role of HA in the pathogenesis of insulin resistance^104^. Furthermore, an HA synthesis inhibitor attenuated NASH-mediated liver fibrosis^106^ (Fig. 4).

The differential abundance of the proteins regulated by Gα_13_ in the liver was determined using proteomics-based approaches^39^. Among the liver-enriched secretory proteins, ITIH1 was revealed as a key molecule associated with metabolic defects. ITIH1, an HA-binding protein (i.e., SHAP–HA complex), is predominantly expressed in hepatocytes under diabetic conditions. Several studies have demonstrated changes in ITIH1 levels under pathological situations^107^. ITIH1 levels were decreased in patients with hepatic fibrosis^108^. Circulating ITIH1 mRNA levels were elevated in rats with D-galactosamine-induced liver injury. Bleomycin affects ITIH1 expression in a lung fibrosis model^109^. Hepatic Gα_13_ levels were diminished under diabetic conditions in which ITIH1 was the major driver of organ cross talk controlled by the liver. ITIH1 is overexpressed in the absence of hepatic Gα_13_, secreted through the circulation and directly binds to HA in the adipose tissue and skeletal muscle to stabilize the integrity of each, thereby aggravating peripheral insulin resistance. Hepatic Gα_13_ increased ITIH1 overexpression through *O*-linked β-N-acetylglucosamine transferase-catalyzed *O*-GlcNAcylation. The Gα_13_-mediated signaling cascade evident in systemic glucose intolerance provides a new conceptual framework implicating the liver as the primary metabolic organ critical for whole-body glucose metabolism under diabetic conditions.

The roles of Gα_13_ in energy metabolism differ substantially in the skeletal muscle (prodiabetic) and liver (antidiabetic)^39,40^. GNA13 (encoding Gα_13_) is predominantly expressed in muscles compared with its expression in other metabolically active organs^40^. Exercise diminished Gα_13_ expression but increased Gα_12_ expression^39^. Deficiency of Gα_13_ in muscles causes skeletal muscle to acquire the oxidative phenotype, and the accompanying reciprocal increase in Gα_12_ promotes fatty acid oxidation. Koo et al. found that muscle-specific deficiency of Gα_13_ protected mice from diet-induced adiposity with increased fatty acid metabolism. Because intramyocellular lipids are primary contributors to insulin resistance, muscle-specific deficiency of Gα_13_ enhances muscle glucose metabolism and insulin sensitivity^40^. In contrast, deficiency of Gα_13_ in the liver does not affect glucose metabolism in the liver but causes overproduction of ITIH1 in hepatocytes. Circulating ITIH1 consequently binds to HA on the surface of adipose tissue and skeletal muscle, culminating in systemic insulin resistance^39^.

Interestingly, Gα_12_ is also present in the endoplasmic reticulum and serves as an activator of the endoplasmic reticulum export machinery^110^. The COPII subunit Sec24 senses cargo folding and acts as a GEF to activate Gα_12_^110^. Activation of Gα_12_ in endoplasmic reticulum exit sites responding to a folded cargo protein load facilitates cargo export and suppresses protein synthesis; this process is also known to autoregulate endoplasmic reticulum export (AREX)^110^. AREX signaling regulates a fraction of the secretome^110^.

### Role of Gα_12_ in liver fibrosis

Under the condition of liver fibrosis, HSCs are activated and transdifferentiated into myofibroblasts, which produce aberrant ECM in response to liver injury^111^. HSCs are activated by mediators such as platelet-derived growth factor (PDGF) and transforming growth factor-beta (TGF-β). Certain GPCR pathways promote liver fibrogenesis. Levels of thrombin, lysophosphatidic acid, endothelin-1, sphingosine-1-phosphate, angiotensin II and acetylcholine are all elevated during liver fibrosis, and most GPCRs activated by these ligands are coupled to Gα_12_. Moreover, among the G protein members, Gα_12_ is particularly overexpressed in activated HSCs^45^. Thus, signals from activated GPCRs in the HSCs are augmented. Gα_13_ is not significantly affected in these cells.

The transdifferentiation of HSCs and the resultant changes in ECM proteins are also controlled by miR-29b, miR-150, and miR-194, suggesting the pleiotropic action of multiple microRNAs on HSC activation. As a liver-enriched miRNA, miR-16 has been shown to affect Bcl-2 and cyclin D1 to control HSC proliferation and apoptosis resistance^112–114^. In addition, miR-16 dysregulation contributes to the activation of HSCs through Gα_12_ overexpression (Fig. 4).

Autophagy is the key process for organelle turnover and nutrient recycling. Under nutrient deprivation, autophagy is initiated. As a result, metabolites and macromolecules such as amino acids, glucose, fatty acids and nucleic acids are made available as energy sources. Dysregulation of autophagy is often associated with metabolic diseases, including obesity, diabetes and cardiac diseases. Impaired autophagy results in triglyceride accumulation and insulin resistance in the liver^115^. Autophagy regulates beige adipocyte maintenance and adipocyte differentiation^116^ and controls glucose tolerance and muscle mass^117^. Therefore, the dysregulation of autophagy inhibits adipocyte differentiation and muscle atrophy. Autophagy supplies the energy necessary to support HSC transdifferentiation by mobilizing lipids and inducing mitochondrial oxygen consumption, which allows HSCs to cope with energy demands and maintain phenotype and cell homeostasis. Hence, autophagy may be an important modulator of signaling pathways in HSCs. Autophagy is accompanied by changes in ATG5/12, consistent with reports that ligands known to activate GPCRs coupled with Gα_12_ (e.g., thrombin, sphingosine-1-phosphate and angiotensin II) stimulate autophagy. Because ATG5/12 are the key mediators in late stage autophagy^118^, it is inferred that Gα_12_ plays a role in the signal amplification during autophagy in HSCs and that its dysregulation contributes to liver fibrosis.

Previously, JNK was identified as a kinase regulated by the Gα_12_ signaling pathway during different biological events^62^; further, JNK activation promotes α-SMA expression in response to TGF-β, PDGF and angiotensin II, thereby activating HSCs. The Gα_12_-mediated JNK pathway participates in multiple autophagy steps, such as ATG12-5 conjugation. Gα_12/13_ increased Rho/Rac-dependent AP-1 activity. In another study, Gα_12_ signaling enhanced Nrf2 ubiquitination and degradation^119^. Nrf2 may be a promising target for the suppression of HSC activation; this possibility is supported by the finding that liver injuries caused by toxicants promote HSC activation through increased oxidative stress and/or decreased Nrf2. Moreover, Nrf2 activation may elicit an antifibrotic effect by inhibiting TGF-β/Smad signaling^114^. Together, HSC activation induced by Gα_12_ overexpression may be associated with increased Nrf2 degradation and TGF-β/Smad pathway activation.

### Role of Gα_12_ in cardiovascular disease

Metabolic syndrome is defined as a combination of cardiovascular risk factors associated with obesity, diabetes, dyslipidemia and hypertension. Gα_12_ plays a role in the cardiovascular system. Baseline blood pressure is regulated by Gα_q/11_ but not Gα_12/13_^58,120^. However, salt-triggered hypertension is dependent on the Gα_12/13_ signaling pathway^121^. The vasoactive compound lysophosphatidic acid is a blood-derived bioactive lipid. Lysophosphatidic acid promotes transient hypertension mainly via the Gα_12/13_-coupling LPA4 receptor, one of five LPA receptors (LPA1–4 and LPA6). The ROCK inhibitor Y-27632 successfully suppresses LPA-induced hypertension, suggesting that LPA increases blood pressure via the Gα_12/13_-Rho/ROCK pathway^122^. Angiotensin II is an important factor for increasing blood pressure via AT1. In vascular smooth muscle cells, AT1-Gα_12_ activates Rho/ROCK in the rostral ventrolateral medulla in the brain. Inhibition of Gα_12_ via oligodeoxynucleotide infusion attenuated angiotensin II-mediated hypertension^123^. While the angiotensin II AT1 receptor antagonist has an anti-hypertensive effect in both young and old animals, the endothelin ET_A_ antagonist darusentan can reduce blood pressure in aged animals. Endothelin increases the vascular tone in the smooth muscle of aged mice in a Gα_q/11_- and Gα_12/13_-dependent manner^124^.

Under conditions of vascular injury, the hyperplastic proliferation of vascular smooth muscle cells occurs and causes neointimal hyperplasia, a condition of exaggerated intimal thickness. The GPCR ligand sphingosine-1-phosphate stimulates abnormal vascular smooth muscle cell proliferation by inducing the secretion of ECM-associated proteins, particularly cysteine-rich protein 61 (CYR61). CYR61 is a member of the connective tissue growth factor family. Sphingosine-1-phosphate regulates Gα_12/13_-Rho-dependent CYR61 induction, leading to hyperplastic vascular abnormalities^125^. Thromboxane A2 can also induce vascular smooth muscle cell proliferation and migration. The thromboxane A2 receptor activates Yes-associated protein (YAP)/transcriptional coactivator with PDZ-binding motif (TAZ) to activate Gα_12/13_ and thus enhance these processes^126^. The Hippo pathway inhibits YAP/ TAZ, which sense mechanical cues, such as ECM stiffness^127^. During atherogenesis, the lysophosphatidic acid levels are increased, which then activates YAP/ TAZ through Gα_12/13_-coupled receptors^128,129^. Integrins are noncanonical Gα_13_-coupled receptors that mediate cell-ECM adhesion. Gα_13_ directly binds to integrin β3 and regulates integrin outside-in signaling^125^. Upon unidirectional shear stress, integrin is activated, leading to an interaction between integrin and Gα_13_^130^. This interaction inhibits RhoA and YAP/TAZ activity, thereby delaying atherogenesis.

Pressure overload induces cardiac fibrosis. Mechanical stretching enhances the release of nucleotides such as ATP and UDP from cardiac myocytes via pannexin-1. Nucleotides outside the cell control the mechanical stretch-induced activation of Gα_12/13_ through the P2Y6 receptor. Inhibition of Gα_12/13_-coupled P2Y6 receptors lowers fibrogenic factors and angiotensin-converting enzyme levels, inhibiting cardiac fibrosis^131^. P2Y6 receptors heterodimerize with AT1 receptors. The formation of AT1R-P2Y6 receptor heterodimers enhances vascular hypertrophy and angiotensin II-induced hypertension^132^.

## Concluding remarks

**Fig. 5:**
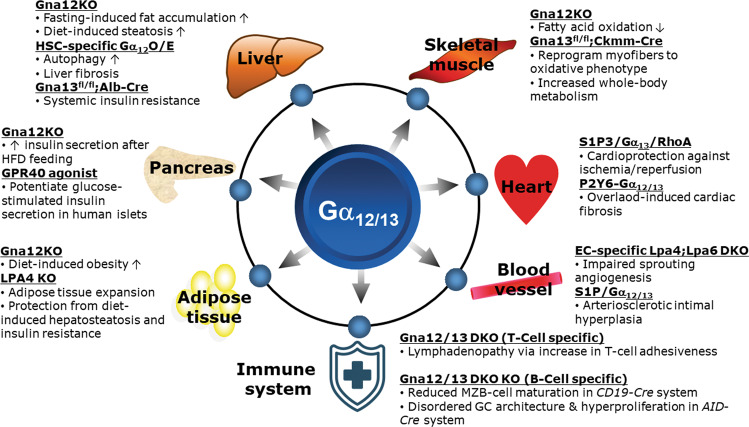
Overview of the roles of Gα_12/13_ signaling in different metabolic organs. The roles of Gα_12/13_ and Gα_12/13_-coupled receptors, such as G-protein-coupled receptor 40 (GPR40), lysophosphatidic acid receptor (LPA4/6), sphingosine-1-phosphate receptor (S1P3), and the purinergic P2Y6 receptor in metabolic organs, the cardiovascular system, and the immune system, are summarized. Gna12-knockout (KO) mice showed enhanced fasting-induced fat accumulation and diet-induced steatosis in the liver and insulin secretion after high-fat diet (HFD) feeding in the pancreas and promoted an increase in fat mass but decreased fatty acid oxidation in skeletal muscle. Liver-specific Gna13-KO mice developed systemic insulin resistance, whereas skeletal muscle-specific Gna13-KO mice showed myofiber reprogramming and thereby increased whole-body metabolism. T cell-specific Gna12/13-double-knockout (DKO) mice had lymphadenopathy, whereas B cell-specific Gna12/13-DKO mice showed reduced marginal zone B cell (MZB cell) maturation and GC architecture.

Diverse activation pathways in many GPCRs converge through heterotrimeric G proteins. In contrast to the mechanisms of GPCR regulation, the regulatory mechanisms of G proteins have not been completely elucidated. For more than 25 years, Gα_12_ has been of great interest in the field of cancer biology. Gα_12/13_ play multifunctional and distinct roles at multiple stages in different organs and in the development of metabolic diseases. In pathological states, abnormal expression of GPCR ligands, Gα_12/13_-coupled receptors and G proteins is often observed. G proteins are important mediators that transduce the signals through GPCRs to intracellular secondary messengers, leading to cellular responses. The regulation of GPCRs by phosphorylation and ubiquitination and the consequent degradation of GPCRs have been extensively analyzed. The cell-targeted gene delivery system and phenotyping of cell-specific knockout mice revealed novel roles for Gα_12/13_ (Fig. 5). Based on their pathological role in most prominent tissues and cells, Gα_12/13_ are associated with obesity, glucose intolerance, hepatic steatosis and cardiovascular disease, and the GPCR–Gα_12/13_ axis is considered an attractive biomarker and therapeutic target for the diagnosis and treatment of metabolic diseases. We suggest that Gα_12/13_-coupled receptors or downstream effectors may be of use as druggable targets. For example, ITIH1 antibodies can be developed for the treatment of type 2 diabetes. In several studies, it has been shown that targeting GPCR-G protein signaling pathways may provide opportunities to overcome certain metabolic diseases. A better understanding of the ligand-receptor-G protein signaling network may provide us with new strategies and methods for the prevention and treatment of metabolic diseases.
